# A machine learning ensemble approach to predicting factors affecting the intention and usage behavior towards online groceries applications in the Philippines

**DOI:** 10.1016/j.heliyon.2023.e20644

**Published:** 2023-10-04

**Authors:** Ma Janice J. Gumasing, Ardvin Kester S. Ong, Madeline Anne Patrice C. Sy, Yogi Tri Prasetyo, Satria Fadil Persada

**Affiliations:** a School of Industrial Engineering and Engineering Management, Mapúa University, Philippines. 658 Muralla St., Intramuros, Manila, 1002, Philippines; b E.T. Yuchengo School of Business, Mapúa University. 1191 Pablo Ocampo Sr. Ext., Makati, Metro Manila 1205, Philippines; c Department of Industrial Engineering and Management, Yuan Ze University, 135 Yuan-Tung Rd., Chung-Li, 32003, Taiwan; d Entrepreneurship Department, BINUS Business School Undergraduate Program, Bina Nusantara University, Jakarta 11480, Indonesia

**Keywords:** Online groceries, UTAUT2, PMT, Random forest classifier, Artificial neural network

## Abstract

The emergence of e-commerce platforms, especially online grocery shopping, is heightened by the COVID-19 pandemic. Filipino consumers started to adapt online due to the strict quarantine implementations in the country. This study intended to predict and evaluate factors influencing the intention and usage behavior towards online groceries incorporating the integrated Protection Motivation Theory and an extended Unified Theory of Acceptance and Use of Technology applying machine learning ensemble. A total of 373 Filipino consumers of online groceries responded to the survey and evaluated factors under the integrated framework. Artificial Neural Network that is 96.63 % accurate with aligned with the result of the Random Forest Classifier (96 % accuracy with 0.00 standard deviation) having Perceived Benefits as the most significant factor followed by Perceived Vulnerability, Behavioral Intention, Performance Expectancy, and Perceived. These factors will lead to very high usage of online grocery applications. It was established that machine learning algorithms can be used in predicting consumer behavior. These findings may be applied and extended to serve as a framework for government agencies and grocers to market convenient and safe grocery shopping globally.

## 1 Introduction

**Fig. 1:**
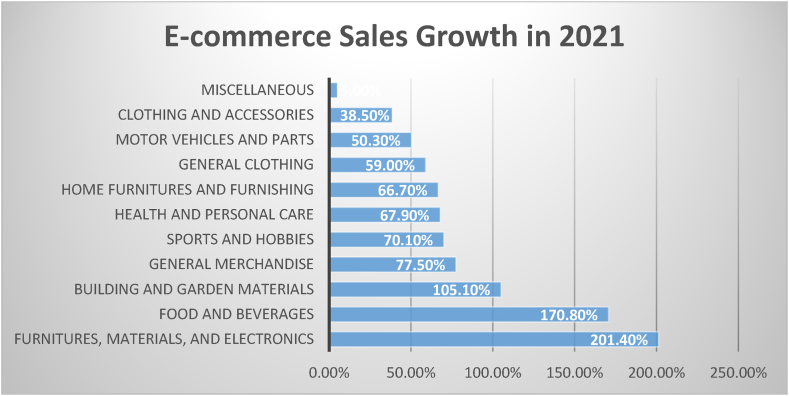
E-Commerce growth rate.

Electronic commerce or e-commerce provides a platform to individuals or organizations who buy and sell goods and services online [1]. As the digital age continuously advances, it has also significantly altered the business sector due to the widespread availability of internet-ready devices such as laptops, smartphones, or tablets where consumers are able to instantly shop. The rapid emergence of e-commerce integration serves as an effective driver of economic development projected in the substantial growth rate of the Asian economy, such as China, with predictions of further progression [2]. Fig. 1 represents the e-commerce growth rate in 2021, wherein furniture, building materials, and electronics ranked first. Second, it was seen that food and beverages increase 170.8 %, and building materials, garden equipment and supplies ranked third [3]. In Fig. 1, the x-axis represents the percent growth, while the y-axis corresponds to the area of e-commerce sales. The increase has been sought to be underexplored, specifically in the consumer side dealing with their intention to use online shopping mobile applications.

In China and India, it was found that e-commerce markets increased by 27 % and 75 %, respectively – as to why the market trend in the Asia-Pacific region is deemed to have the fastest development [4]. In relation with the economic development manifested in the Asia-Pacific region, Chen and Kimura [5] iterated that there is a $1.6 trillion recorded revenue from international e-commerce platforms in 2018 and is forecasted to reach $2.7 trillion by 2023. The Philippines took part in the advancement of e-commerce, highlighting the rise during the COVID-19 pandemic. Prasetyo et al. [6] showed how food delivery applications were on the rise during the pandemic in the Philippines, positing how the country led to an increase in technology engagement. Despite being a developing country, the citizens of the Philippines rapidly got accustomed to the use of technology and its development [7]. This led to an increase in their technology consumption and consideration of available platforms for ease and efficiency, especially in the food sector advancement of technology.

The food sector is regarded as one of the industries facing difficulties to be digitized as there are requirements in terms of expiry dates, regulations, and supplier deal alterations since consumers are expecting fresh food to be provided upon purchase [8]. In the advent of online shopping, Khatwani [9] found that the majority of the respondents in India opted to shop traditionally rather than digitally due to unsatisfactory experience, delivery options, and policies regarding return and exchange. On the other hand, Melis et al. [10] determined how multi-channel retailers affect the experience of consumers in the United Kingdom, where multi-channel retail mix is the provision of online and offline options when shopping in the retail industry. Findings show that the unfamiliarity of the dynamics of an online grocery became a significant constraint of customers when shifting from an offline to online channel. However, upon constant use and becoming accustomed to the online platform, consumers start to favor online groceries over traditional groceries. Another issue distinguished by Jilcott Pitts et al. [11] as to why customers hesitate to switch to online groceries includes uncertainty with perishable items, which leads to increase in unhealthy impulse purchases of food options with preservatives. Leaning towards the use of online groceries, Collison [12] foresaw that the food sector will thrive through the acceleration of online food delivery services due to its manifestation of expeditious expansion in the past few years, in which it may contribute to a wider acceptance of online groceries. As seen in the study of Prasetyo et al. [6], Filipinos got quickly accustomed to using technology in food sector industries due to its usability and efficiency.

The change of e-commerce platforms from traditional to e-commerce was seen in the boost of global e-commerce revenue by 52 % in China, 41 % in South Korea, 7 % in France, 5 % in Japan, the United States, 8 % in the United Kingdom, and 12.93 % in the Philippines [13]. With that, all major grocery stores in the United Kingdom have started to offer online channels as early as 2018 and escalated during the COVID-19 pandemic due to the evident increase in sales, reaching $15.4 billion from online groceries alone, which is not yet as evident in the Philippines [14]. When it comes to the local e-commerce scene in the Philippines, it is in line with preceding finding wherein the grocery section has been receiving an increasing amount of attention in e-commerce platforms in 2018 [15] but has been underexplored. The Philippine online grocery market does not only feature big supermarket chains, but also third-party applications or marketplaces which offer above satisfactory service, delivery, and product variety.

It was seen that habits of individuals are affected, which mainly involve spending and eating practices. The sudden shift to a pandemic setting made consumers indulge in panic buying which was supported by a finding by Ferreira Rodrigues et al. [16] stating that the consumers in Brazil are acquiring a larger amount of food through online food delivery service, which contrasts pre-pandemic eating habits. Changes in attitude and habits are due to people having a decrease in confidence with their choice to shop traditionally, which in turn instilled hesitancy since threats of a contagious disease and government restrictions are present [17].

Initially, online groceries were meant for individuals who do not have sufficient time to personally shop for their necessities. However, having experienced the pandemic, Filipinos have adjusted to the new normal in which online buying such as groceries have become important to the consumer's end since risks are reduced [18,19]. The traditional set-up of groceries have been indicated to increase the spread of COVID-19, different supermarkets and marketplaces in the Philippines have entered the e-commerce, such as GrocerGenie PH, All Day Market, GrabMart, PandaMart, Lazada, Shopee, San Miguel Foods, Inc., Landers Superstore, WalterMart Supermarket, Lucky Mart, MerryMart, Pick.A.Roo, SM Markets, MetroMart, and Robinsons Supermarket [20]. Provided that several online groceries offer home deliveries in the Philippines, there is still a need to further explore and understand the behavioral intention of customers to avail services from the grocers.

In the past years, numerous studies have tackled behavioral intention of consumers on the use of online groceries around the world. Gutama and Dewi Intan [21] examined the acceptance of consumers towards a digital supermarket in Indonesia utilizing the basic Technology Acceptance Model (TAM). It was found that perceived usefulness, perceived ease of use, and social influence significantly affect consumer acceptance wherein perceived usefulness yielded the largest statistical effect. To further widen the scope of understanding the customer adoption of online groceries, Ryadi et al. [22] also conducted an analysis in the same country using an extended version of TAM, inclusive of trust and perceived risk as additional constructs. Contrary to findings in the aforementioned literature, perceived usefulness was the only construct crucial in the behavioral intention to use e-groceries. While TAM is proven to be an effective theoretical measure of usage intention, several academic works have used supplementary variables due to the limitations brought by the original model [23].

Due to the constraints seen in TAM, Venkatesh et al. [24] proposed the Unified Theory of Acceptance and Use of Technology (UTAUT2) as an avenue to unify characteristics of older theories and models based on corresponding strengths and weaknesses. Upon comparing, it was found that four (4) core constructs, namely performance expectancy, effort expectancy, social influence, and facilitating conditions, provide a more thorough assessment of behavioral intention and usage behavior [25,26]. Three constructs, such as price value, hedonic motivation, and habit were added by Venkatesh et al. [27], which then became known as UTAUT2 and generated a solid advancement in the assessment of behavioral intention and system usage [28,29]. Hence, UTAUT2 is widely used to determine behavioral intention and usage of emerging systems which involve novel online applications including e-groceries.

This model has been used and expanded in a study conducted in Belgium. Van Droogenbroeck & Van Hove [30] investigated the intention of consumers to repurchase grocery items electronically using the UTAUT2 model with four (4) additional constructs such as perceptions of time pressure, enjoyment in shopping, risk, and service quality. It was identified that innovativeness and the perception of time pressure significantly contribute to the behavioral intention of non-users in utilizing online supermarkets; while habit and performance expectancy are deemed as significant influencers of the behavioral intention to repurchase and utilize groceries online. Moreover, Çakir & Kazançoğlu [31] also broadened the UTAUT2 model by integrating perception risks specifically time, psychological, physical, and social risks to cater the personal preferences of consumers in terms of behavioral intention to adopt online groceries in Turkey. Unlike other studies, habit, facilitating conditions, time risk, and psychological risk are components that yielded a statistically valid influence on behavioral intention to use online groceries. Despite this, psychological risk showed a negative relationship with the behavioral intention. Additionally, a study utilized UTAUT2 and added perceived risk and perceived trust to the framework to analyze customer intention to opt for online grocery shopping [32]. Findings reveal that performance expectancy, price value, hedonic motivation, and habit have a direct relationship with the behavioral intention to purchase grocery items online.

It is evident that existing studies have varying results on which components drive the behavioral intention of consumers to use online groceries. Chang et al. [29] linked discrepancies in results of the UTAUT2 model to diverse external components such as culture or technological advancement in different countries. In relation, another context that affects the behavioral intention of individuals to use online groceries is the psychological effect of the COVID-19 pandemic. A study in Thailand examined factors that influence customers’ behavioral intention and actual usage of e-groceries using the UTAUT2 framework in consideration of the global health crisis evident during the COVID-19 virus [33].

Several studies that tackle the acceptance of the use of online groceries are present, but there are inconsistencies with existing results due to the inclusion of constructs beyond the UTAUT2 offers, which in turn provides a subjective take on the behavioral intention to use e-groceries especially in a pandemic setting. Thus, it is essential to incorporate consumer preference theories that are health-centered in nature. Given that, the Protection Motivation Theory (PMT) could be considered as an extension upon the integration with UTAUT2. Chuenyindee et al. [28] considered the integration of PMT to assess the technology acceptance of people in using contact tracing applications in Thailand. It was seen that PMT as an integration can holistically measure behavioral intentions to use an application when health concerns are in play. Similarly, the study of Ong et al. [29] also considered integrating PMT with another theory due to health-related studies. Thus, it justifies how PMT, and its constructs could be used to measure health-related behaviors of individuals [30].

While studies evaluating factors influencing behavioral intention of customers to adopt online groceries are available across the globe, there is a lack of holistic measurement of behavioral intentions in using a technology when health-related concerns are present. As explained from the study of Gumasing et al. [25], relatively, no studies in the Philippines have quite established the online grocery acceptance, people's behavioral intention, and its overall usage. Thus, this study aimed to predict factors affecting the behavioral intention and usage behavior among consumers on online groceries during the course of the COVID-19 pandemic utilizing the integrated PMT and UTAUT2. Several literatures commonly used the multivariate statistical tool to assess human behavior. However, Woody [36] criticized this method due to the presence of indirect effect and mediator available in a model. This is also supported by Fan et al. [37], thus the need to utilize a more substantial method to cater to the disadvantage brought by the SEM could be utilized. Ong [38] presented how a machine learning ensemble may be used to completely measure behaviors among individuals. Their study considered the ensemble of artificial neural network and random forest classifier which produced a high accuracy rate for prediction. Therefore, this study utilized the two algorithms to predict factors influencing usage behavior of consumers towards online groceries.

The results of this study would be beneficial to grocery retailers who intend to maximize their opportunities by adding an online channel for their customers. In addition, the first study that considered MLA could be applied and extended to justify the claim that using these tools could measure behavioral intentions [30,38]. This study will also be of aid to venture capitalists and administrators upon strategizing the marketing of online groceries to customers. Furthermore, the results can provide a framework among future researchers who cover e-commerce, behavioral aspects among consumers, and for developers worldwide.

## 2 Conceptual framework

**Fig. 2:**
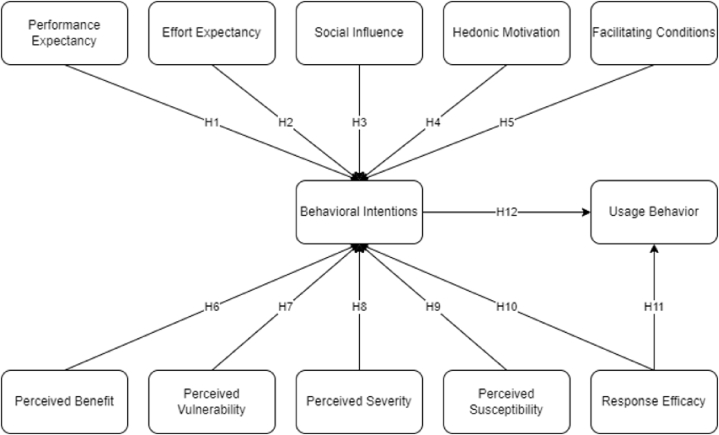
Conceptual framework.

The proposed conceptual framework for this study depicted in Fig. 2 is based on the extended UTAUT2 with the integration of PMT. Several constructs were assessed in this study simultaneously such as: performance expectancy, effort expectancy, social influence, hedonic motivation, and facilitating conditions under UTAUT2. Moreover, the PMT constructs such as perceived benefits, perceived vulnerability, perceived severity, perceived susceptibility, and response efficacy were considered to measure behavioral intention to use online groceries during the COVID-19 pandemic.

Performance expectancy is defined as the degree to which the utilization of newly introduced technology will help target users to gain benefits upon use [27]. This implies that the improvements in performance observed by users will increase the likelihood of them to perceive online groceries as beneficial in their end. Zhou et al. [39] found that performance expectancy is the major indicator of behavioral intention to use live e-commerce shopping which is in line with the findings of Chang et al. [40] when it comes to the use of online shopping. Additionally, it can be further traced in the study by Lee et al. [41] that performance expectancy serves as an important component in the continuous intention to use food delivery applications. Thus, it was hypothesized that.

H1 Performance expectancy has a positive and significant effect on behavioral intentions.

Effort expectancy is characterized by the level of effort a user needs to exert when using a novel technology, specifically online grocery applications [24]. Davis [42] introduced this construct in the TAM, in which it was explained that a system that is easier to learn and use, the more it is accepted by users. With that said, Hungilo [43] highlighted that effort expectancy is the one of the top factors affecting behavioral intention of shoppers to purchase their items digitally. This finding is supported by Rasli et al. [44], in which it was distinguished that there is a positive and significant influence of effort expectancy to the behavioral intention of consumers to utilize online food delivery options. Thus, it was hypothesized that.

H2 Effort expectancy has a positive and significant effect on behavioral intentions.

Social influence indicates that behavioral intention to use digital supermarkets is affected by the view or opinions of other people, such as family or friends [45]. Venkatesh et al. [24] described this construct with subjective norms such as social factors, rather than individualistic factors. This indicates that a decision of a user to adopt novel technology based on the perceptions from social groups is a driver of behavioral intention. Given that, this claim is supported by the findings of Abdullah [46] as the behavioral intention of users to pursue digital transactions where social influence has a significant contribution. In an online shopping context, Usman Muhammad Umar [47] proved that the viewpoints of family members, colleagues, and peers majorly affect the behavioral intention of users. Given these findings, it has been hypothesized that.

H3 Social influence has a positive and significant effect on behavioral intentions.

One's sense of enjoyment experienced upon using technology, such as e-groceries, which aid in the encouragement of individuals to adopt a new system could be assessed with the hedonic motivation latent variable [48]. With this construct, utilitarian ideologies are being satisfied due to the unveiling of emotional and multisensory factors of the target [49]. Several studies proved that hedonic motivation contributes to the users' intention to use new technology, such as Ravangard et al. [50] as the behavioral intention to use medical laboratory portals was investigated. The findings of Nikolopoulou et al. [51] further validates the importance of hedonic motivation in the study examining the acceptance of the adoption of mobile internet. Thus, it was hypothesized that.

H4 Hedonic motivation has a positive and significant effect on behavioral intentions.

Facilitating condition covers the availability of organizational and technical infrastructures upon completing a transaction using online grocery applications [24]. It is described how facilitating conditions are essential due to its ability to reduce, if not remove, barriers when using new systems [28]. Alqahtani & Braun [52] supports the claim of the existing studies since results show that facilitating conditions is proven to have a positive effect on the behavioral intention when it comes to cybersecurity compliance. In addition, Saprikis et al. [53] identified that facilitating conditions as a direct and major determinant of behavioral intention to adopt mobile augmented reality shopping applications. To which.

H5 Facilitating condition has a positive and significant effect on behavioral intentions.

Perceived benefits pertain to the discernment of an individual on how a course of action, with opting for online groceries rather than traditional groceries amidst the global health crisis as an example, will reduce the risk of a disease and lead to positive results [54]. In a pandemic context, perceived benefits are associated with acting upon COVID-19 preventive measures, such as wearing a mask, practicing social distancing, and following quarantine restrictions [55]. Similarly, the use of online apps for grocery instead of going to mortar and bricks store regarded beneficial to prevent COVID-19 infection. It was indicated from the study of Walrave et al. [56] how perceived benefit of application utilized during the COVID-19 pandemic affected behavioral intentions significantly. Similar to the study of Ong et al. [34] and Chuenyindee et al. [28]. Thus, it was hypothesized that.

H6 Perceived benefits has a positive and significant effect on behavioral intentions.

Perceived vulnerability covers the unfavorable attributes linked to a recommended health action to avoid the COVID-19 infection [55]. This may be due to the lack of technological literacy to operate online channels of groceries, lack of encouragement for usage, or low priority based on the definition of perceived barriers in a study tackling behavior of women regarding breast cancer screening [57]. Moreover, Ong et al. [34] highlighted how perceived vulnerability is an important factor needed to be considered when evaluating acceptance of people upon utilizing a technology. Their study indicated how perceived vulnerability and perceived benefits are key attributes whether an individual will be open to use and accept a technology. Thus, the formulated hypothesis is.

H7 Perceived vulnerability has a positive and significant effect on behavioral intentions.

Perceived severity is defined by the level of seriousness an individual believes a health issue is. This is viewed as the degree of adverse outcomes caused by actions that are disease-related such as COVID-19 [58]. Sreelakshmi & Prathap [59] proved that perceived severity is a major indicator of continuous adoption of mobile e-payments during this time of pandemic to prevent physical contact. This was validated by a study that assessed perceptions of the public on social distancing where it was found that perceived severity majorly drives people to prefer alternatives that allow them to adhere to minimum health standards [60]. Thus.

H8 Perceived severity has a positive and significant effect on behavioral intentions.

Perceived susceptibility is identified as the level of vulnerability of an individual to a disease [61], especially senior citizens, people with comorbidities, and pregnant women who have higher risk of experiencing severe symptoms of COVID-19 [62]. Shewasinad Yehualashet et al. [63] emphasized that perceived susceptibility is a major indicator of COVID-19 preventive measure adoption as people believe that they are susceptible to the virus, even though they do not belong to the high-risk priority groups set by the government. In an e-commerce context during the COVID-19 pandemic, Pang et al. [64] found that perceived susceptibility is a significant predictor of intention to use livestream shopping technology since consumers find traditional shopping risky due to the virus threat. Thus, it was hypothesized that.

H9 Perceived susceptibility has a positive and significant effect on behavioral intentions.

Response efficacy are external prompts that encourage a person to pursue a health-driven course of action [65]. In a study to analyze mobile health applications, Melzner et al. [66] explained that although a person initially intends to adopt health-related technology, there is still a need to be reminded in order to act upon the intention. Thus, response efficacy is a significant factor that should be considered when designing application features to promote recommended health actions to prevent the spread of COVID-19 virus. Furthermore, Karimy et al. [67] showed how intention of individuals to adopt COVID-19 preventive measures through the utilization of mass media and sending notifications in mobile numbers and social media to practice minimum health standards. Moreover, Chuenyindee et al. [38] highlighted how response in using mobile applications related to COVID-19 prevention, in which online groceries are a part of is increased when people perceive the health-related benefits. Thus, it was hypothesized that.

H10 Response efficacy has a positive and significant effect behavioral intentions.

H11 Response efficacy has a positive and significant effect on consumers' usage behavior.

Behavioral intention is derived from the model formulated by Davis et al. [42]. This construct has been established to have a positive relationship with the actual usage behavior of users towards new technology. Zahra et al. [68] confirmed that behavioral intention entails the continuous use of technology in the future. With that said, several studies associated behavioral intention to the usage behavior of technologies that promote contactless transaction especially during the COVID-19 pandemic with the goal of practicing social distancing and reducing the likelihood of being infected [[69], [70], [71], [72]]. Therefore, it was hypothesized that.

H12 Behavioral intention has a positive and significant effect on consumers' usage behavior.

The overall hypotheses build up, relationships, and supporting references are summarized in Table 1.

**Table 1:** Summarized hypotheses build-up.

Hypotheses	Relationship	References
1	Performance expectancy → Behavioral intention	[27,[39], [40], [41]]
2	Effort expectancy → Behavioral intention	[24,[42], [43], [44]]
3	Social influence → Behavioral intention	[24,[45], [46], [47]]
4	Hedonic motivation → Behavioral intention	[[48], [49], [50], [51]]
5	Facilitating Conditions → Behavioral intention	[24,28,52,53]
6	Perceived benefit → Behavioral intention	[28,34,[54], [55], [56]]
7	Perceived vulnerability → Behavioral intention	[34,55,57]
8	Perceived severity → Behavioral intention	[[58], [59], [60]]
9	Perceived susceptibility → Behavioral intention	[[61], [62], [63], [64]]
10	Response efficacy → Behavioral intention	[[38,[57], [58], [59], [60], [61], [62], [63], [64], [65]]]
11	Response efficacy → Usage behavior	
12	Behavioral intention → Usage behavior	[42,[68], [69], [70], [71], [72]]

## 3 Methodology

### 3.1 Research paradigm

Reviewing the article published by Rahi [73], this study prompted to consider a positivist paradigm since an established theory was implemented for evaluation. The research was centered on employing an experimentation to create an observation for usage behavior of online grocery applications in the country. With the evaluation of the integrated theories, the current study evaluated the preceding factors using an experimentation process through the optimization of creating a classification model through machine learning ensemble. The output presents an observation as to why users have a positive or negative intention to utilize online grocery applications. As support, this paradigm has been established to be stable at the recent observation as output are circled around the objective measurement made by researchers [74]. The theory focuses more on the deductive level as the UTAUT2 and PMT are highly established theories utilized in their respective fields of technology evaluation and health protective motivations. Since fresh data were collected and evaluated using established theories, Collis et al. [75] justified how the deductive level can support the assumptions made. In relation to this study, the hypotheses created were the assumptions created and evaluated through quantitative method. In addition, the convenience sampling approach was considered in this study as it was explained to be cost-effective and efficient [73]. However, since bias may be present, only those who are and have used online grocery applications were considered in this study. In addition, the population size was considered similar to the explanation of Cochran [73]. This study employed Yamane Taro calculation as common practice in technology acceptance evaluation and health protection motivation studies employed this technique. Moreover, item development through the use of literature review was considered. The build up of the measure items in this study considered adapted question items of studies that utilized the same framework and latent variables – evaluated using a 5-point Likert Scale survey. Presented in the succeeding sections are the specifications of the different areas to evaluate the overall objectives of the study.

### 3.2 Respondents

This study considered 373 valid responses among online grocery mobile application users. This generated a total of 21,261 data points which were processed through data cleaning and data aggregation to feed as input to the Python Integrated Development Environment – Spyder 5.0. Through convenience sampling, the data were collected online through different social media platforms due to the protocols set by the COVID-19 pandemic. The minimum sample size required for this study is 271 based on the study by Adam [76] on the work of Yamane Taro. Using this sample size, the margin of error is 5 % at a significance level for categorical data with a population size of greater than 100,000. Prior to dissemination, the consent form among respondents were collected. Moreover, the survey was approved by Mapua University Research Ethics Committees (Document No.: FM-RC-22-30). The response was collected by distributing the online survey in different social media platforms, made available from August 2021–December 2021.

### 3.3 Questionnaire

An adapted 67-item questionnaire [25] that were categorized in four (4) sections with respect to the conceptual framework that integrated the UTAUT2 and PMT was considered in this study. The first section of the questionnaire establishes the demographic profile of the target respondents of this study. The second part of the questionnaire covers UTAUT2 indicators with 23-item questions derived from existing studies. The third part of the questionnaire included the indicators from the PMT, with 34-item questions. This part is allotted for the measurement of the coping and threat appraisal and usage of online groceries with the consideration of the COVID-19 pandemic. Each question utilized a 5-point Likert Scale [6,30,38,69,77,78].

Lastly, an 8-item questionnaire completed the items which aided in the evaluation of behavioral intention and usage behavior of the respondents towards using online grocery applications. Similar to the other parts, the questions for this section were answered using a 5-point Likert Scale ranging from strongly disagree to agree. The measures that determined the behavioral intention and usage behavior were based on the studies conducted by Refs. [30,69,76].

### 3.4 Data pre-processing

**Fig. 3:**
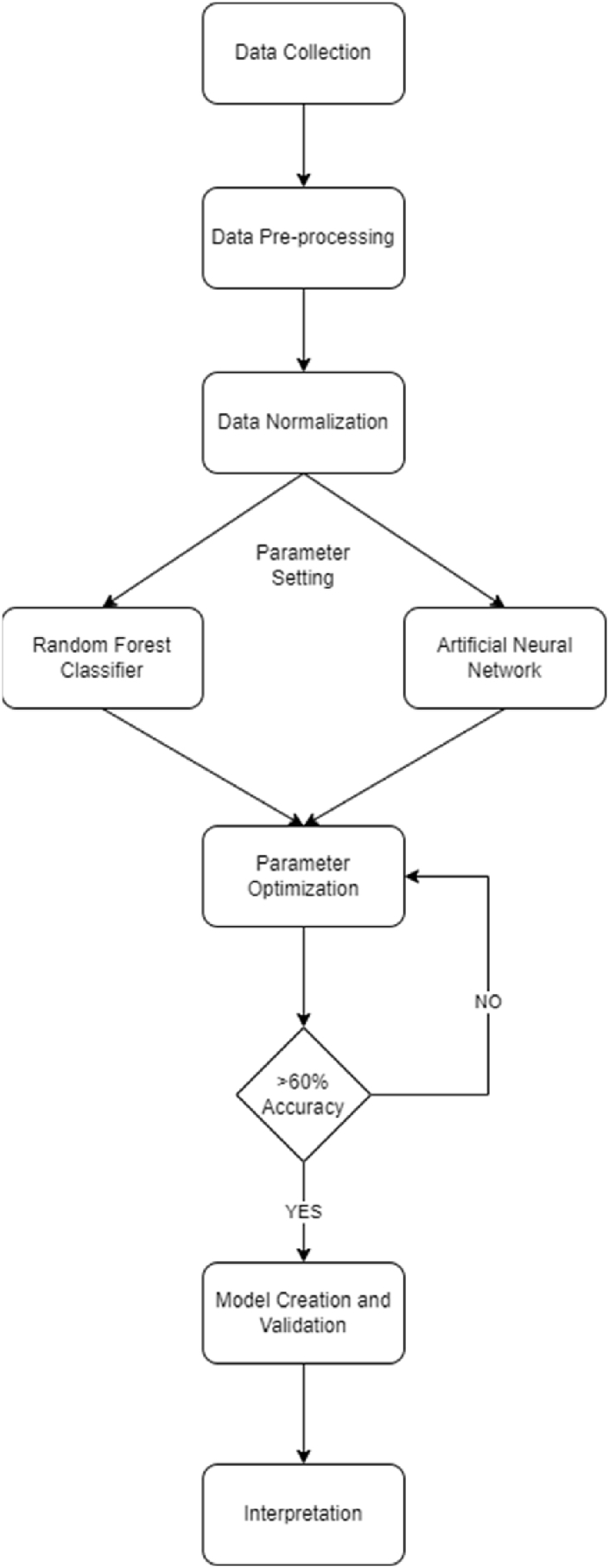
Methodological flowchart.

Following different studies [38,78], the 21,261 data points underwent data cleaning using correlation analysis. A threshold of 0.20 correlation coefficient with p-value 0.05 was considered for significant indicators. Following which is data aggregation where the mean of significant indicators represented the latent variables. The python package for normalization (min_max scalar) employed. Python Integrated Development Environment – Spyder 5.0 was utilized in this study with SKLEARN and TENSORFLOW packages for running the machine learning algorithms. After which, respective optimization for the ensemble was performed. Presented in Fig. 3 is the methodological flowchart of the detailed process.

### 3.5 Random forest classifier

Random Forest Classifier is a classification tool that is used in determining significant factors dealing with human behavior [79]. Chen et al. [79] explained that Random Forest Classifier produced higher accuracy compared to other types of decision tress, with higher classification rate. Similarly, Gao et al. [80] considered the same algorithm for classification because it unifies the dataset and produces a powerful and reliable output. Compared to the simple decision tree, the Random Forest Classifier can split the leaves with the best branch [81]. Thus, employing Random Forest Classifier was considered to determine significant latent variables affecting the usage behavior on online groceries during the COVID-19 pandemic.

A total of 6400 runs were considered in this study in optimizing the Random Forest Classifier. In an attempt to generate the optimum tree, the parameters such as entropy and gini, together with the splitters, best and random, and tree depth from 4 to 7 with different training and testing ratios, 60:40–90:10 were considered. A total of 100 runs per combination was conducted and analyzed using Analysis of Variance (ANOVA) for significant difference test.

### 3.6 Artificial neural network

After data pre-processing, the aggregated data underwent optimization for running the Artificial Neural Network. It is a supervised machine learning algorithm tool that mirrors the biological neural system through the utilization of artificial neurons [82]. The neural network mechanism is mimicked through various mathematical functions that is able to extract patterns in large datasets in a feed-forward manner [83]. Artificial Neural Network requires information from the input layer which are considered the artificial neurons, the aggregated data. To which, these are processed to identify the non-linear relationship present in an analysis which passes through the hidden layer and results to the output layer. German et al. [78] explained that it promotes analysis of nonlinearly correlated variables accustomed to human behavior analysis. Similarly, Ong et al. [34] resulted with great accuracy in the Artificial Neural Network process for analysis of behavioral intention among technology adoption in Thailand.

Based on several literatures that used machine learning algorithm to predict behavior shown in Table 2, the activation functions considered for the hidden layer were ReLu [84,85], Sigmoid [[84], [85], [86]], and Tanh [87]. Moreover, the activation functions considered for the output layer was Softmax [84,88,89]. Lastly, the optimizers that were used in the initial optimization were Adam [85,87,[89], [90], [91]] and RMSProp [90,91]. A total of 10 iterations per combination was employed at 150 epochs [92] with consideration of hidden layer nodes.

**Table 2:** Artificial neural network parameters.

Hidden Layer Activation Function	References
ReLu, Sigmoid, Softmax	[84]
Sigmoid, ReLu	[85]
Sigmoid	[86]
ReLu	[[89], [90], [91]]
Tanh	[87]
**Output Layer Activation Function**	**References**
Sigmoid, Softmax	[84]
Softmax	[88,89]
**Optimizer**	**References**
RMSProp, Adam	[84]
RMSProp, Adam, SGD	[90]
Adam	[87,89,91]

## 4 Results

### 4.1 Participants

From the collected data, majority were female (61 %) and the rest were male (39 %) from age range between 31 and 40 years old (34 %), 41–50 years old (25 %), 21–30 years old (25 %), 20 years old and below (14 %), and the rest were older. To which, most of the respondents finished college or with a graduate degree (52 %) or have attended college (31 %) living in the city (62 %). Gumasing et al. [25] explained how most of the people buying grocery among different households were female with children, thus explains how the majority of the respondents were with this gender and age group.

In addition, most of the respondents were seen to be living with 5 or more family members (52 %) or around 3–4 members (39 %) with monthly household income of 100,001–130,000 PhP (30 %), 40,001–70,000 PhP (32 %), earning more than 130,000 PhP (22 %), or earning around 70,001–100,000 PhP (16 %). Similar to the study of Gumasing et al. [25], income ranging from 70,000 PhP and below would be low-income classes (32 %), 70,000–100,000 PhP as middle-income class (16 %), and above 100,000 PhP are high income classes (52 %). Moreover, frequency of buying grocery among respondents was seen to be around twice a month (43 %), once a month (24 %), once a week (24 %), or less than once a month (9 %) with monthly grocery expense of 8001–11,000 PhP (26 %), 2001–5000 PhP (21 %), 5001–8000 PhP (20 %), or 14,000 PhP and above (13 %). The descriptive statistics are presented in Table 3.

**Table 3:** Demographic characteristics.

Factor	Characteristics	n	%
Age	20 years old and below	52	14
	21–30 years old	93	25
	31–40 years old	127	34
	41–50 years old	94	25
	51 years old and above	7	2
Sex	Male	146	39
	Female	227	61
Education	Attended Grade School	26	7
	Attended High School	37	10
	Attended College	116	31
	Finished College and/or Graduate Degree	194	52
Residence	City	231	62
	Province	142	38
Household Members	1–2 people	34	9
	3–4 people	94	39
	5 or more people	145	52
Frequency of Buying Grocery	At least once a month	34	9
	Once a month	90	24
	Twice a month	160	43
	Once a week	89	24
Average Expenditure on Grocery	Around 2000 PhP or less	34	9
	2001–5000 PhP	78	21
	5001–8000 PhP	75	20
	8001–11,000 PhP	97	26
	11,001–14,000 PhP	41	11
	Above 14,000 PhP	48	13
Monthly Total Household Income	Less than 40,000–70,000 PhP	119	32
	70,001–100,000 PhP	60	16
	100,001–130,000 PhP	112	30
	More than 130,000 PhP	82	22

### 4.2 Random forest classifier

Random Forest Classifier has been considered in this study to determine the significant factors affecting the usage behavior on online groceries during the COVID-19 pandemic. After the optimization process, it was seen that depth 6 produced the highest average accuracy with 96 % and 0.00 standard deviation. This shows how the tree produced a consistent result throughout all runs. Presented in Table 4 is the summary of decision tree from Random Forest Classifier mean accuracy result including the F1-Score.

**Table 4:** Decision tree Mean accuracy (depth = 6).

Category	60:40	F1	70:30	F1	80:20	F1	90:10	F1
Random								
**Gini**	93.62	94.03	92.85	93.12	92.88	93.16	92.03	92.87
**Std. Dev**	1.352	1.205	1.872	1.963	4.351	3.720	3.170	3.267
**Entropy**	93.07	93.26	92.79	93.25	93.72	93.98	92.04	92.90
**Std. Dev**	2.440	2.017	1.642	1.557	2.095	2.447	2.733	3.036
**Best**								
**Gini**	94.58	95.31	94.55	95.64	95.52	96.02	**96.00**	**96.00**
**Std. Dev**	0.497	0.864	0.500	0.542	0.502	0.518	**0.000**	**0.000**
**Entropy**	93.60	93.94	91.53	92.08	93.00	93.69	93.00	93.00
**Std. Dev**	0.494	0.883	0.502	0.533	0.000	0.517	0.000	0.000

**Fig. 4:**
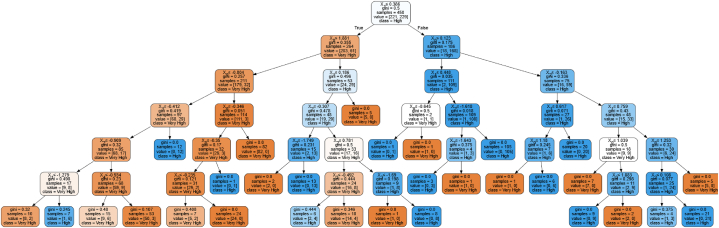
Decision tree from random forest Classifier Legends: X0 – perceived benefit, X1 – perceived vulnerability, X2 – behavioral intention, X3 – performance expectancy, X4 – perceived severity.

Utilizing ANOVA, no significant difference was seen among results, thus this study opted to use the highest accuracy with the lowest standard deviation. It was seen that gini as the criterion and best as splitter at 90:10 training and testing ratio produced the highest accuracy. Fig. 4 represents the optimum decision tree from Random Forest Classifier to determine factors affecting the usage behavior.

Seen in Fig. 4, perceived benefit (X_1_) is the parent node which would dictate factors affecting the usage behavior on online groceries during the COVID-19 pandemic. To which, it will consider the child node, perceived vulnerability (X_0_). Considering the condition of less than or equal to 1.881, X_0_ with value of −0.804 will be in consideration. Not satisfying, X_0_ will be considered and performance expectancy (X_3_) – leading to very high usage behavior of online grocery. This indicates that the higher perceive vulnerability among users for the health effect brought by the COVID-19 pandemic; and how efficient and effective buying grocery online is, users will more likely utilize the mobile application compared to going to stores physically. Nonetheless, if −0.804 is satisfied, it will consider X_1_, behavioral intention (X_2_), and perceived severity (X_4_) which will lead to very high usage behavior on online grocery application due to the foreseen protection for COVID-19 exposure. In addition, not satisfying the first child node will consider X_1_, X_2_, and X_3_ which will lead to high usage behavior on online grocery applications. This result posits that the benefit in using the technology outweighs the perceive vulnerability of health effects and exposure to the COVID-19 virus.

Following the Random Forest Classifier produced, the false from the first node considers X_0_. Either satisfying the condition will consider X_1_ and X_2_ leading to high usage behavior on online grocery applications. Therefore, it could be deduced that X_0_ and X_3_ will lead to very high usage behavior on online grocery applications while X_1_, X_2_, and X_4_ will lead to high usage behavior on online grocery applications. It was expounded from the study of Chen [4] how e-commerce has developed and increase in rate among developing countries. In addition, the U.S Department of Commerce [93] presented the increase in revenue of e-commerce markets. Moreover, Chen and Kimura [5] recorded and forecasted the increase in e-commerce sales to reach approximately 1.7 times higher in 2023. Thus, it justifies how the usage behavior on online grocery is relatively very high due to the foreseen perceived benefits and performance expectancy. To further rank the importance value and most significant latent variables affecting usage behavior on online grocery application, Artificial Neural Network was utilized in this study.

### 4.3 Artificial neural network

**Fig. 5:**
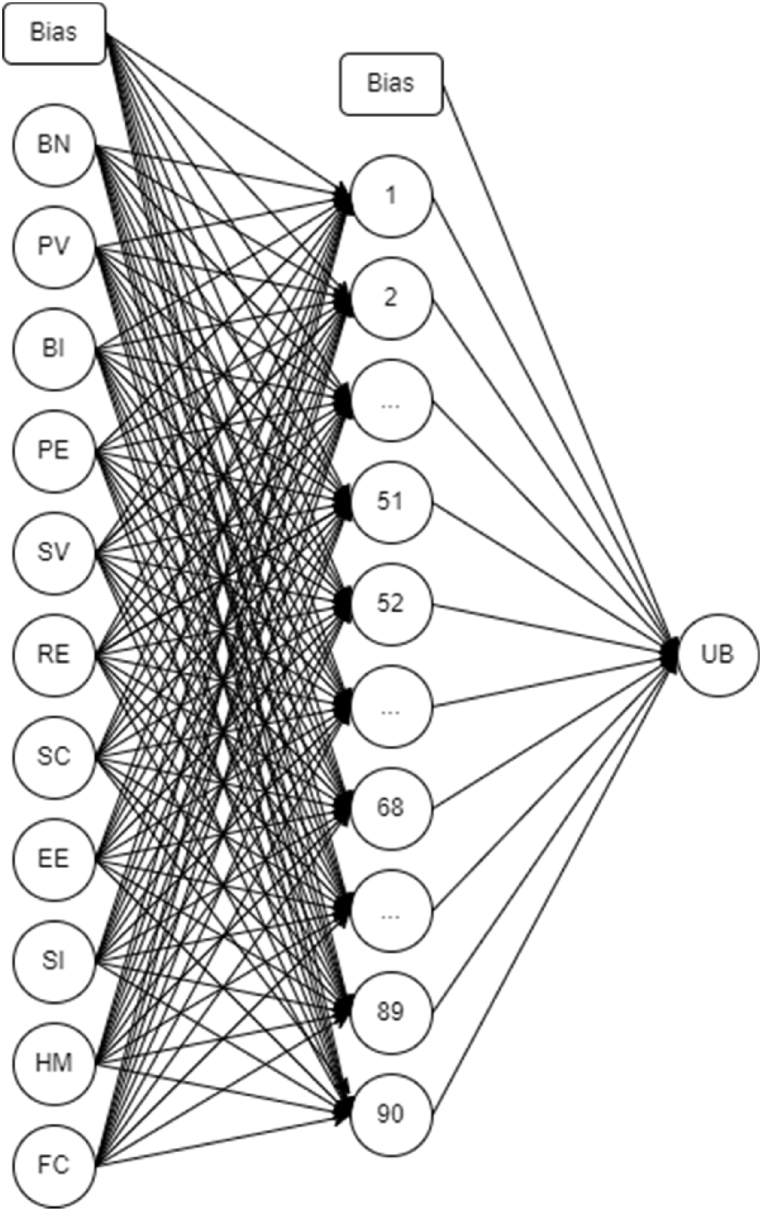
Optimum artificial neural network model.

Artificial Neural Network has been considered in this study to determine the significant factors affecting the usage behavior on online grocery. With the optimization process, it was seen that parameters such as Tanh and Softmax for the activation function of the hidden and output layers, respectfully produced a high accuracy rate (Fig. 5). In addition, Adam was used as the optimizer for the 80:20 training testing ratio set. At 150 epochs and 90 hidden nodes, the average accuracy of 96.63 % was seen with no overfitting was seen from the final Artificial Neural Network model. Presented in Table 5 is the summary of average accuracy for training and testing of the different input considered in this study. Ong [38] indicated that the higher the average accuracy of testing would lead to the most significant factor.

**Table 5:** Summary of results.

Latent	Average Training	StDev	Average Testing	StDev
Perceived Benefit	**89.32**	**1.234**	**95.14**	**1.006**
Perceived Vulnerability	89.26	2.035	94.31	1.326
Behavioral Intention	89.23	1.475	92.86	2.151
Performance Expectancy	88.63	1.965	90.67	2.647
Perceived Severity	85.17	2.248	89.93	3.248
Response Efficacy	83.38	1.898	84.76	1.796
Perceived Susceptibility	74.92	3.664	79.86	1.658
Effort Expectancy	72.26	4.856	77.24	2.487
Social Influence	70.22	4.524	76.87	3.685
Hedonic Motivation	68.48	3.658	73.22	1.032
Facilitating Conditions	60.23	1.126	61.04	1.433

To further validate the results, the score of importance was run to categorize the sequential influence of factors affecting online grocery application usage behavior. In addition, the Cronbach's alpha to test the reliability of the constructs were analyzed. From the results presented in Table 6 , it could be seen that the perceived benefit is the most influential factor, followed by perceived vulnerability, behavioral intention, performance expectancy, perceived severity, response efficacy, perceived susceptibility, effort expectancy, social influence, hedonic motivation, and the least is facilitating conditions. In accordance, a test for multicollinearity was assessed using the variance inflation factor with threshold of 5. All latent variables produced values less than the threshold, indicating no multicollinearity [94].

**Table 6:** Score of importance.

Factors	Importance	Score (%)	Cronbach's Alpha	VIF
Perceived Benefit	0.270	100	0.950	2.907
Perceived Vulnerability	0.265	98.2	0.939	3.638
Behavioral Intention	0.258	95.7	0.887	1.462
Performance Expectancy	0.248	91.8	0.866	3.184
Perceived Severity	0.244	90.3	0.918	4.945
Response Efficacy	0.225	83.4	0.953	2.673
Perceived Susceptibility	0.213	78.9	0.925	4.896
Effort Expectancy	0.189	70.1	0.866	2.682
Social Influence	0.182	67.3	0.899	2.367
Hedonic Motivation	0.164	60.6	0.953	2.660
Facilitating Conditions	0.158	58.6	0.818	1.604

**Fig. 6:**
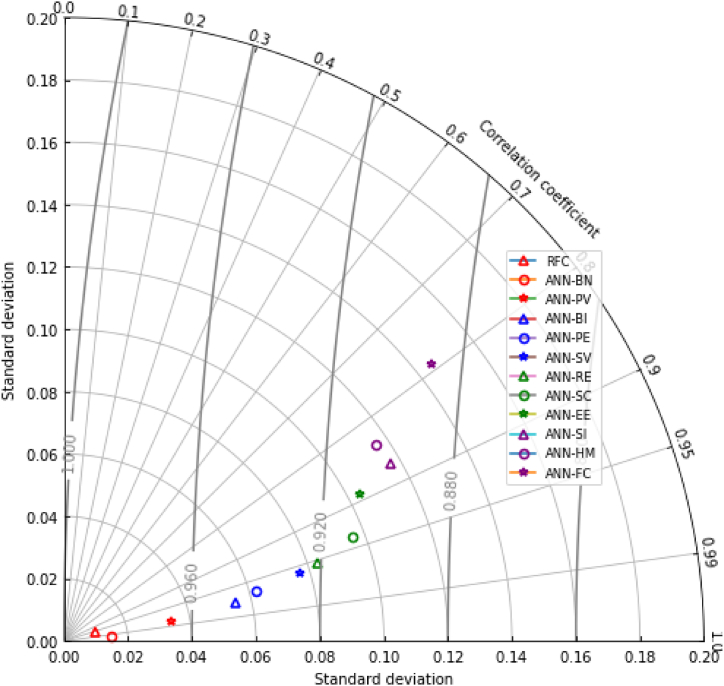
Taylor diagram.

Lastly, the consideration of the Taylor Diagram was considered to further test the validity and accuracy obtained among the different machine learning algorithm utilized in this study. Following the study of German et al. [35,75], the Taylor Diagram is employed to undermine the consistency and compare the accuracy results of the different machine learning algorithm utilized in the study. Expressed by Gholami et al. [95], the Taylor Diagram considers the correlation and standard deviation of the machine learning algorithm results, relating all of which for better validity output. To which, a threshold of 20 % root-mean-square(?) error at 90 % correlation output with 1.0 as standard deviation would present a consistent and acceptable machine learning ensemble. As evident in Fig. 6, all results present consistent output which indicates valid and accepted ensemble utilized in this study.

## 5 Discussion

There is a major shift in demand for online food delivery services upon the global COVID-19 outbreak, which specifically includes online groceries [96]. This is affected by the fear of being infected by the virus as explained by Grashuis et al. [97]. They expounded that customers opt for home delivery service of grocery stores in locations where the virus is spreading at an increasing rate. Additionally, Jilcott Pitts et al. [98] iterated that the emergence of online groceries during the COVID-19 pandemic is a convenient means to acquire grocery goods while adhering to minimum health standards to curb the spread of the virus. Moreover, it also influences people to become health conscious as online groceries prevent impulse buying of food that can be detrimental to the overall health.

In line with that, this study aimed to predict factors influencing the behavioral intention of Filipinos to use online groceries amidst the COVID-19 pandemic utilizing Artificial Neural Network and Random Forrest Classifier. Several constructs under the UTAUT2 and PMT were considered and evaluated simultaneously. The classification model yielded an average accuracy of 96.63 % from the artificial neural network. Furthermore, the random forest classifier generated an average accuracy with 96 % and 0.00 standard deviation.

Ten (10) constructs were analyzed in which perceived benefits were found to have the highest significant influence, thereby supporting H6. Table 7 presents a summarized output of the accepted hypotheses due to significance. From the findings, it is an indication that consumers are more likely to opt for online grocery platforms to lessen their chances, severity, and the possible complications of being infected by COVID-19. Moreover, users also tend to use online groceries to avoid physical contact with other people and being exposed in public places which allows them to follow preventive measures and government restrictions during the COVID-19 pandemic. This is supported by the study of Shah et al. [99], wherein it was found that BN mainly drive citizens from Pakistan to adopt COVID-19 preventive actions. When it comes to an e-commerce context, Xu et al. [100] established that perceived benefits influence Chinese citizens’ intention to participate in online shopping. Thus, highlighting and expanding the benefits of using online groceries will draw more users to use this method of grocery shopping.

**Table 7:** Summarized hypotheses output.

Hypotheses Number	Variable Relationship	Decision
H1	Performance expectancy → Behavioral intention	Accepted
H2	Effort expectancy → Behavioral intention	Accepted
H3	Social influence → Behavioral intention	Accepted
H4	Hedonic motivation → Behavioral intention	Accepted
H5	Facilitating Conditions → Behavioral intention (close to 60 %)	Relative Acceptance
H6	Perceived benefit → Behavioral intention	Accepted
H7	Perceived vulnerability → Behavioral intention	Accepted
H8	Perceived severity → Behavioral intention	Accepted
H9	Perceived susceptibility → Behavioral intention	Accepted
H10	Response efficacy → Behavioral intention	Accepted
H11	Response efficacy → Usage behavior	Accepted
H12	Behavioral intention → Usage behavior	Accepted

Perceived vulnerability is found to be the next most significant factor, thereby supporting H7. This shows that people find it difficult and lack the patience to observe recommended health actions to avoid being infected when shopping at traditional grocery stores. This includes frequent handwashing with soap and water, not touching their hands, lips, nose, or eyes, and wearing of face shield, as to why they opt for online grocery platforms [101]. Aside from that, consumers choose online groceries since purchasing items for sanitary purposes can be costly. It can be drawn that consumers fear being infected by COVID-19 as to why they highly consider actions that may lead to unfavorable outcomes. To which, online groceries offer a safer method for the public to buy their necessities. This also implies that customers who recognize their level of vulnerability during the COVID-19 pandemic are those who are less likely to go to brick-and-mortar stores [102]. To support the findings, several studies have concluded that perceived vulnerability has a positive and significant relationship with the intention to follow COVID-19 cautionary measures [82], which involves securing food supply digitally [103,104].

Behavioral intention places as the third significant construct that influences the usage behavior of consumers towards online groceries, thereby supporting H12. It is an indication that Filipino consumers intend to use online channels of grocery stores to keep themselves, family members, and acquaintances protected from COVID-19. Given that, the influence of behavioral intention to the usage of online groceries amidst the COVID-19 pandemic suggests that consumers aim to mitigate any potential risks brought by the virus [105]. Behavioral intention is deemed to be a major indicator of usage behavior when adopting new technology [28], which includes online groceries in accordance with existing literature [30,106].

Performance expectancy is also found to be a significant determinant of the behavioral intention to use online groceries since consumers are able to procure grocery items faster through this channel, thereby supporting H1. Customers also find that using online platforms for groceries saves time which makes it convenient since the need to worry about the opening and closing time of physical stores when buying goods and necessities is eliminated. This is in line with the findings of several studies [107,108] wherein performance expectancy is the major contributor to the BI to adopt online shopping even before the COVID-19 pandemic occurred. In a pandemic context, the influence of performance expectancy to the behavioral intention to use e-commerce platforms inclusive of food delivery services was further heightened as seen in the works of Muangmee et al. [109] and Vinerean et al. [110]. Given that this construct has a high level of importance, it must be taken into consideration that grocers should improve the state of their online channels.

Perceived severity has been proven to have a significant impact on the intention and actual usage of online groceries, thereby supporting H8. Results suggest that people fear the consequences of being infected by COVID-19 covers such as high mortality rate and increasing transmission rate even though the target vaccination rate for the population in the Philippines has been attained [111]. This may be due to the history of the Philippines having the highest daily cases in Southeast Asia in 2020 in which the pandemic response is deemed subpar as manifested in the Bloomberg's COVID-19 resilience ranking, in which the country had placed last and only climbed up a few places [112]. Given that context and even though the government quarantine restrictions are currently lax in the Philippines, people are still cautious due to the degree of possible negative outcomes being infected by COVID-19 entails. To which, it urged them to prefer contactless transactions that allow them to stay at home instead of going out in public – which online groceries offer. This is supported by Pang et al. [64] wherein it was found that perceived severity positively and significantly impacts the consumption behavior of people towards online livestream shopping. Additionally, Güngördü Belbağ [113] concluded that perceived severity of COVID-19 strongly affects the preference of consumers when buying essential goods as it was found that people avoid physical stores to lessen the likelihood of transmission and lockdown – which can also be applied in the Philippines.

Response efficacy was found as the next most significant construct that affects the behavioral intention and usage behavior towards online groceries, thereby supporting H10 and H11. This means that the use of online grocery shopping is a result of external factors such as government-mandated policies and restrictions to curb the transmission of the virus along with the recommendation of peers and family members in order to protect one's health. Griffin et al. [114] have identified that response efficacy is a core driver of protective behavior against COVID-19, which means that it increases the likelihood of people preferring options that decrease the risk of being infected by the virus, such as online groceries instead of brick-and-mortar retail stores. Having said that, risk communication campaigns also serve as effective external prompts to urge the public to follow COVID-19 precautionary actions in Ethopia [115]. Similar results were also seen in the study of Tomczyk et al. [116] wherein response efficacy influences the behavioral intention and actual usage behavior due to the stimulation of protective behavior. To further support that, Ezati Rad et al. [103] stated that preventive behaviors against COVID-19 are warranted by triggers which makes RE an indicator of motivation for adopting these actions.

Perceived susceptibility is deemed to be a significant contributor to the behavioral intention to use online groceries during the COVID-19 pandemic, thereby supporting H9. This is an indication that consumers using online groceries are driven by their subjective view regarding the level of exposure they have in terms of virus transmission along with the possible complications being infected involves. Existing studies support this claim in which perceived susceptibility is an indicator of contactless food services usage [117]. However, contrasts with Gumasing et al. [25] in which perceived susceptibility was proven to be insignificant which may be associated with the demographic profile of the respondents and the Delta variant being the dominant variant during the duration of the study. It can be deduced that customers take necessary preventive measures to stray away from situations that leave them vulnerable to the virus due to the soar of COVID-19 cases, especially during the Omicron variant surge in the Philippines wherein it reached more than 30,000 cases per day in January 2022 as it affects any individual regardless of age [118,119].

Effort expectancy has been proven to have a significant impact on the BI to use online grocery platforms, thereby supporting H2. It can be deduced that the level of effort needed to be exerted by consumers to use online groceries is generally acceptable, in such a way that the system is simple to use and there are no technical issues encountered. This is supported by the study of Kappor and Singh [120], wherein it was found that effort expectancy is positively correlated to the BIto adopt mobile commerce. In addition, Chayomchai et al. [121] concluded that effort expectancy is a major determinant to use technology, which includes e-commerce platforms during quarantine period. Innovations such as online groceries aid to combat the impacts of the COVID-19 [122]. Given that this study considered a third-world country, the use of technological innovation is gradually progressing. It is therefore essential that novel technology is accessible and easy to learn for all users.

Social influence was found to be a significant determinant of usage behavior towards online groceries, thereby supporting H3. This finding suggests that the preference to use online groceries during the COVID-19 pandemic is impacted by the opinions of family members, friends, community members, and other acquaintances. Contrary to existing literature, social influence has been regarded as insignificant in terms of continuous use of online food delivery applications in Jordan and e-commerce in Slovenia [123,124]. However, Zanetta et al. [125] highlighted the significance of social influence on the behavioral intention to use food delivery applications in Brazil during the COVID-19 pandemic. From there, it can be drawn that the risk perception of one's social circle can manifest as protective behavior that may lead them to recommend the use of technology, such as online groceries, to protect themselves against COVID-19 exposure and transmission.

Hedonic motivation was also found to have a significant impact on the behavioral intention to use online grocery platforms, thereby supporting H4. This indicates that the pleasure and satisfaction when using online groceries positively affect the usage behavior of Filipino consumers amidst the global health crisis. Findings suggest that consumers find online groceries delightful and comfortable to use in comparison to traditional grocery stores aside from the benefit of being less exposed to the virus. In relation to that, Vinerean et al. [110] determined hedonic motivation as the strongest contributor to customer satisfaction and intention to mobile commerce. To further heighten this, Zanetta et al. [125] associated the significance of hedonic motivation with the satisfaction encountered in terms of availability, familiarity, and overall experience when using online food delivery services.

Facilitating conditions was found to be the last significant predictor of behavioral intention, thereby supporting H5, but has relatively low significance. This is an indication that consumers have the essential resources and skills that enables them to shop for grocery items online during the COVID-19 pandemic, which encourages them to use the said platform. Many studies have concluded that facilitating conditions is not significant when it comes to adopting contactless transactions and mobile shopping platforms since smartphone applications are generally easy to operate and internet connectivity is accessible for other countries [126,127]. However, Leong et al. [128] stated that facilitating conditions is a significant indicator of behavioral intention to use online transactions in developing countries such as the Philippines. Thus, improving the facilitating environment can increase the likelihood of consumers using online groceries in the country.

Overall, the usage of online groceries during the COVID-19 pandemic was observed to be majorly affected when Filipino consumers grasp the benefits brought by online groceries, the efficiency of using this platform, their level of vulnerability to the COVID-19 virus, and the severity of symptoms once infected. Thus, perceived benefits, perceived vulnerability, behavioral intention to use, performance expectancy, and perceived severity would lead to very high and high usage of online groceries in the Philippines amidst the global health crisis. Given that, more consumers, even in Third World countries, appear to become more accepting of contactless transactions and online platforms of traditional stores. Therefore, the findings of this study can provide a basis with regard to the future of online groceries, due to its convenience, performance, and benefits even after the pandemic.

### 5.1 Theoretical Implication

Numerous studies that tackle indicators of purchasing behavior in the e-commerce industry during the COVID-19 pandemic have been conducted as it is essential in order for businesses to determine which course of action should be implemented to satisfy the needs of consumers. The evaluation of consumer behavior and acceptance of online groceries in the Philippines in the course of the global health crisis using machine learning ensembles presented many strengths when it comes to behavior prediction and which factors contribute to it. Artificial neural network is one of the algorithms used in this study in which it is preferably used for pattern recognition as it emulates the biological component of neurons in the brain [129]. This is commonly used to classification of big datasets, time series forecasting, and function approximation or regression [130]. When it comes to prediction, artificial neural network has been found to have a higher level of predictive and classification power when compared to statistical methods such as function approximation or regression since it provides a multi-layer analysis of complex datasets [131].

Given that context, Niazkar & Niazkar [132] utilized artificial neural network to forecast the confirmed, recovered, and death cases of COVID-19 in several countries across the globe which include China, Japan, Singapore, Iran, Italy, South Africa, and the USA. This was done to formulate health-related policies that are aligned with the data pattern available. Similarly, Çaparoğlu et al. [133] investigated the efficiency of COVID-19 mitigation policies in Turkey in which it was found that the strategies implemented along with the actions concerning vaccination and potential mutations of the virus is pivotal in containing the spread of the virus. Thus, artificial neural network aids in the determination of policies and strategies including their effectiveness during the COVID-19 pandemic.

On the other hand, random forest classifier is also used in this study as another machine learning algorithm. Biau et al. [134] stated that this decision tree-based algorithm is one of the most accurate classifiers available and generates reliable outputs. Moreover, Cutler et al. [135] enumerated the benefits of random forest classifier which includes the ability to model interactions and handle big and complex datasets. As to why different studies [34,35,38] employed both artificial neural network and random forest classifier to determine what influences the perceived usability of a contact tracing application in Thailand.

In comparison to existing literature, this study provided a new and thorough approach in analyzing consumer behavior which encapsulates the behavioral intention to use and actual usage behavior of consumers of emerging e-commerce platforms due to the COVID-19 pandemic. Having said that, the results of this study exhibit a 96.63 % accuracy level from the artificial neural network which is consistent with the 96 % accuracy level from the random forest classifier results. It can be inferred that the accuracy levels from both machine learning algorithms are reliable and can be used as a framework for future studies under the same discipline.

### 5.2 Practical Implication

Assessing the factors affecting the behavioral intention to use and usage behavior of Filipino consumers towards online groceries during the COVID-19 pandemic is crucial to grocers, business owners, and e-commerce developers in general. The findings of this study indicate that even though the Philippines is a Third World country, Filipinos are open to use online groceries instead of going to traditional stores due to the benefits and efficiency online supermarkets offer. This framework allows grocery retailers to fully understand what drives consumers to opt for online platforms when acquiring grocery items, which in turn provides them opportunities to strategize the development and implementation of an online channel in addition to brick-and-mortar retail stores.

Furthermore, results of this study imply that government agencies may further create risk awareness campaigns and policies to prompt Filipinos to continuously use online groceries as means to follow COVID-19 preventive measures. This is based on the results of this study which suggest that Filipinos opt for alternatives that will mitigate their risks of virus transmission as they further understand their level of vulnerability and the severity of the outcomes once infected by COVID-19. Hence, this will lead to a fuller understanding of the advantages present when choosing to buy necessities through online groceries. In turn, venture capitalists may use this framework to develop a marketing strategy not just in the Philippines, but in other countries as well wherein people still opt for traditional supermarkets.

### 5.3 Limitations

The findings of this study exhibit promising results which can be developed as a framework for future studies concerning consumer behavior. However, there are still areas that can be further evaluated since there are limitations observed in this study. First, respondents who answered the digital survey majorly reside in highly urbanized cities in the Philippines, which may affect their level of technological literacy. Thus, future researchers may consider a greater number of Filipino consumers from rural areas as respondents to provide an authentic representation. Second, there is a lack of consideration of socio-economic factors of the respondents for this study. It is suggested to utilize demographic information including age, sex, household monthly income, educational attainment, and employment status. A clustering technique can be done such as K-Means as a method of customer segmentation. This will extensively identify consumer needs in which grocery retailers can use as a framework to satisfy demand based on the mentioned factors. Thus, these limitations should be addressed in future studies to enhance current findings.

## 6 Conclusion

In the past decade, the e-commerce industry exponentially grew across the globe as access to technology has become prevalent. The COVID-19 pandemic heightened this trend as people are urged to stay at home due to government restrictions to curb the transmission of the COVID-19 virus. To which, the Philippines experienced the longest and strictest quarantine implementation in the world [18]. This circumstance forced Filipino consumers to use online and contactless transactions even for grocery shopping due to the limitations the government-mandated lockdowns come with. Given that, the intention to use and actual usage behavior towards online groceries are needed to be explored.

Upon the employment of artificial neural network, it was found that all ten (10) constructs are significant to the behavioral intention and actual use of online groceries at 150 epochs and 90 hidden nodes with an average accuracy level of 96.63 % and a standard deviation of 0.0367 with no overfitting. This is persistent with the results of the random forest classifier with an average accuracy level of 96 % and a standard deviation of 0.00. It was found that once Filipinos are presented with the advantages and efficiency of online grocery shopping during the pandemic accompanied by the awareness of their vulnerability of being infected by the virus and the severity of the illness once infected will lead to high usage behavior.

Moreover, the use of machine learning ensembles is established to predict consumer and human behavior and technology acceptance due to the high accuracy levels presented in the findings of this study. Hence, the framework of this study can be adopted by future researchers to further study human behavior. In addition, in order to further urge consumers to prefer online groceries, there is a need to ingrain the benefits, efficiency, vulnerability, and severity. Government agencies and grocery retailers may make use of the findings to maximize their opportunities in both COVID-19 prevention and penetration of e-commerce platforms. In addition, this will aid in the collective movement to stop the spread of COVID-19, not just in the Philippines, but also around the world.

## Data availability statement

Data will be made available on request.

## Declaration of competing interest

The authors declare that they have no known competing financial interests or personal relationships that could have appeared to influence the work reported in this paper.
