# Supplementary material for: A machine learning ensemble approach to predicting factors affecting the intention and usage behavior towards online groceries applications in the Philippines
Source: Heliyon. 2023 Oct 4;9(10):e20644. doi: 10.1016/j.heliyon.2023.e20644 (PMC10560843; doi:10.1016/j.heliyon.2023.e20644)
Supplement: Multimedia component 1 [file mmc1.docx]

Indicators for Measurement

| **Construct** | **Items** | **Measure** | **Reference** |
| --- | --- | --- | --- |
| Performance Expectancy | PE1 | I can buy groceries more rapidly when I use online grocery. | Chopdar et al. (2018);  Van Droogenbroeck & Van Hove (2021) |
|  | PE2 | Using online grocery improves my chances of accomplishing more essential goals. |  |
|  | PE3 | I can save much time using online groceries. |  |
|  | PE4 | Online grocery shopping is convenient because it reduces my reliance on store hours. |  |
| Effort Expectancy | EE1 | Online grocery services are simple to use, in my opinion. | Van Droogenbroeck & Van Hove (2021) |
|  | EE2 | I have no trouble finding what I need when using online groceries. |  |
|  | EE3 | It is not difficult to order things from an online grocery. |  |
|  | EE4 | Using an online grocery store, you can quickly check the availability of goods. |  |
| Social Influence | SI1 | My family members believe that ordering groceries online is a great idea. | Prasetyo et al. (2021);  Van Droogenbroeck & Van Hove (2021) |
|  | SI2 | Most of my acquaintances and friends think that buying groceries online is an excellent idea. |  |
|  | SI3 | In my community, shopping for groceries online is a status symbol. |  |
|  | SI4 | People who sway my decisions believe that I should shop for groceries online. |  |
|  | SI5 | People around me think it's perfectly acceptable to shop for groceries online. |  |
| Facilitating Conditions | FC1 | I have the necessary resources to shop at an online grocery store. | Yuan et al. (2014);  Prasetyo et al. (2021); Van Droogenbroeck & Van Hove (2021) |
|  | FC2 | I have the essential skills to shop for groceries online. |  |
|  | FC3 | When I have problems using the online grocery, a specialized person (or group) is accessible to help me. |  |
|  | FC4 | Other technologies I use are compatible with online grocery. |  |
| Hedonic Motivation | HM1 | I find online grocery fun to use. | Yuan et al. (2014);  Prasetyo et al. (2021); Van Droogenbroeck & Van Hove (2021) |
|  | HM2 | I find online grocery enjoyable to use. |  |
|  | HM3 | I find online grocery very entertaining. |  |
|  | HM4 | The use of online grocery amuses me. |  |
|  | HM5 | The use of online grocery makes me feel good. |  |
|  | HM6 | I feel comfortable using online grocery. |  |
| Response Efficacy | RE1 | My family and friends will support me if I shop for groceries online. | Shahnazi et al. (2020);  Yuen et al. (2021) |
|  | RE2 | Because the government strongly encourages me not to go out, I shop for groceries online. |  |
|  | RE3 | I will only buy groceries on-site after COVID-19 if I am given appropriate external information about existing safeguards; as a result, I prefer to shop online. |  |
|  | RE4 | More people are using online groceries this pandemic; thus, I use online grocery. |  |
|  | RE5 | My own experience with online grocery convinced me to use it again. |  |
| Perceived Benefits | BN1 | Using online grocery can reduce my chance of infection; thus, I use online grocery. | Wong et al. (2021); Bechard et al. (2021);  Kamran et al. (2021) |
|  | BN2 | Using online grocery can decrease the severity and the chance of complications if I get infected with COVID-19; thus, I use online grocery. |  |
|  | BN3 | Using online grocery will avoid contact with other people and crowded places; thus, I use online grocery. |  |
|  | BN4 | I want to adhere to the principles of prevention and government restrictions; thus, I use online grocery. |  |
|  | BN5 | I stay at home to control the pandemic sooner; thus, I use online grocery. |  |
| Perceived Vulnerability | PV1 | It is difficult to follow the COVID-19 prevention recommendations; thus, I use online grocery. | Shahnazi et al. (2020) |
|  | PV2 | I don't have the patience to follow COVID-19 precautionary measures; thus, I use online grocery. |  |
|  | PV3 | I find it challenging to repeatedly wash hands with soap and water; thus, I use online grocery. |  |
|  | PV4 | It's tough to avoid touching your hands, lips, nose, or eyes; thus, I use online grocery. |  |
|  | PV5 | Face shield is inconvenient to use and uncomfortable; thus, I use online grocery. |  |
|  | PV6 | I find disinfectant solutions expensive and scarce in the market; thus, I use online grocery. |  |
| Perceived Severity | SV1 | The COVID-19 has a high mortality rate; thus, I use online grocery. | Shahnazi et al. (2020);  Tadesse et al. (2020) |
|  | SV2 | The COVID-19 is very dangerous; thus, I use online grocery. |  |
|  | SV3 | The transmission of COVID-19 is relatively high; thus, I use online grocery. |  |
|  | SV4 | If I am infected with COVID-19, I believe my health will be seriously harmed; thus, I use online grocery. |  |
|  | SV5 | Because of the possibility of contracting COVID-19, I will not go to the hospital if I become unwell with another condition; thus, I use online groceries. |  |
| Perceived Susceptibility | SC1 | I believe I am at risk of COVID-19; thus, I use online grocery. | Jiang et al. (2009); Shahnazi et al. (2020);  Tadesse et al. (2020) |
|  | SC2 | I believe I have a higher chance of contacting COVID-19 than before; thus, I use online grocery. |  |
|  | SC3 | I worry about COVID-19, and I cannot do my daily activities like before; thus, I use online grocery. |  |
|  | SC4 | I can contact COVID-19 if I do not take any preventive measures; thus, I use online grocery. |  |
|  | SC5 | I'm terrified to contact sick people with the flu (e.g., cough, sneezing, runny nose, fever); thus, I use online grocery. |  |
| Behavioral Intention | BI1 | I intend to use online grocery to prevent my infection from COVID-19. | Driediger and Bhatiasevi (2019);  Yuen et al. (2019) |
|  | BI2 | I intend to use online grocery to protect my family from COVID-19 infection. |  |
|  | BI3 | I intend to use online grocery if it becomes widely available in my area. |  |
|  | BI4 | I intend to recommend online groceries to my family and friends for safety during the COVID-19 pandemic. |  |
| Usage Behavior | UB1 | I have used the online grocery app | Chopdar et al. (2018) |
|  | UB2 | I have used different types of online grocery apps. |  |
|  | UB3 | I frequently use online grocery in buying goods. |  |
|  | UB4 | I frequently search for new items or goods on an online grocery app. |  |
